# DEVELOPING AN INTERACTIVE COUNTY MAPPING TOOL TO ADVANCE AGE-FRIENDLY PROGRESS IN NEW JERSEY

**DOI:** 10.1093/geroni/igad104.0709

**Published:** 2023-12-21

**Authors:** Emily Greenfield, Clara Scher, Karen Alexander, Joanne Fetzko, Lucas Marxen, Althea Pestine-Stevens, Davit Topchishvili, Michael Pagan

**Affiliations:** Rutgers, The State University of New Jersey, New Brunswick, New Jersey, United States; Rutgers, The State University of New Jersey, New Brunswick, New Jersey, United States; Voorhees Transportation Center, Edward J. Bloustein School of Planning and Public Policy, Rutgers University, New Brunswick, New Jersey, United States; Somerset County Office On Aging & Disability Services, Somerville, New Jersey, United States; NJAES Office of Research Analytics, Rutgers University, New Brunswick, New Jersey, United States; Rutgers, The State University of New Jersey, New Brunswick, New Jersey, United States; Bergen County Division of Senior Services, Hackensack, New Jersey, United States; Bergen County Division of Senior Services, Hackensack, New Jersey, United States

## Abstract

This presentation will describe how a multidisciplinary team at Rutgers, The State University of New Jersey, leveraged a small, internal grant opportunity to partner with leaders of two County government systems to develop a “proof of concept” age-friendly County mapping tool. Based on the Image:NYC mapping tool, the team designed the tool to display the spatial locations of high concentrations of older adults with shared characteristics (e.g., by health status, income) alongside the spatial locations of assets in local communities (e.g., libraries, senior activity centers) at the census tract level within each of the two counties. The presenters will explain how gerontological expertise—in combination with insights, data, and resources from other disciplines and project contributors—were essential to design, produce, and disseminate the map. The presenters will describe how the mapping tool is one component of a growing body of work across the public, private, and academic sectors in New Jersey to advance and support the age-friendly communities movement. The presenters also will reflect on how the age-friendly mapping tool has served to kickstart age-friendly planning and systems change at the county level, with implications for advancing both state- and municipal-level efforts.

